# Bone mineral density alteration in obstructive sleep apnea by derived computed tomography screening

**DOI:** 10.1038/s41598-022-10313-w

**Published:** 2022-04-19

**Authors:** Sharon Daniel, Yafit Cohen-Freud, Ilan Shelef, Ariel Tarasiuk

**Affiliations:** 1 Sleep-Wake Disorders Unit, Soroka Medical Center, Beer-Sheva, Israel; 2 Radiology Department, Soroka University Medical Center, Beer-Sheva, Israel; 3 Department of Public Health and Pediatrics, Faculty of Health Sciences, Ben-Gurion University of the Negev and Clalit Health Services, Southern District, Beer-Sheva, Israel; 4 Department of Physiology and Cell Biology, Ben-Gurion University of the Negev, Beer-Sheva, Israel; 5 Faculty of Health Sciences, Ben-Gurion University of the Negev, Beer-Sheva, Israel; 6 Sleep-Wake Disorders Unit & Department of Physiology, Faculty of Health Sciences, Ben-Gurion University of the Negev, P.O. Box 105, 84105 Beer-Sheva, Israel

**Keywords:** Physiology, Respiration

## Abstract

The association between obstructive sleep apnea (OSA) and bone mineral density (BMD) is poorly elucidated and has contradictory findings. Abdominal computed tomography (CT) for other indications can provide a valuable opportunity for osteoporosis screening. Thus, we retrospectively explored the association between OSA and BMD by examining abdominal CT vertebrae images for a multitude of conditions and indications. We included 315 subjects (174 with OSA and 141 without OSA) who performed at least two CT scans (under similar settings). Both groups had a similar duration between the first and second CT scans of 3.6 years. BMD decreased in those with OSA and increased age. A multivariate linear regression indicated that OSA is associated with BMD alterations after controlling for age, gender, and cardiovascular diseases. Here, we report that OSA is associated with BMD alterations. Further studies are required to untangle the complex affect of OSA on BMD and the possible clinical implications of vertebra-depressed or femoral neck fractures.

## Introduction

Obstructive sleep apnea (OSA) is a common disorder that is associated with recurrent episodes of airway obstruction during sleep^1,2^. It affects more than one in seven adults^1,2^, many of who are undiagnosed^3,4^. This disorder is associated with a large body mass index (overweight and obesity), metabolic abnormalities, and cardiovascular diseases^1^. OSA has been associated with impaired motor function, increased risk for accidents, low bone mineral density (BMD), and fractures^3,5–10^.

Radiological interpretations of spine computed tomography (CT) scans for other clinical indications are the first to identify low bone density and osteoporosis, with no additional cost or exposure to unnecessary radiation^11–14^. CT examination allows BMD to be measured independent of large body mass index^15,16^. To the best of our knowledge, only one study investigated the association between OSA and CT-derived BMD alterations^17^; Hamada et al.^17^ found that males with severe OSA had alterations in lumber vertebra BMD.

Little information is available on the association between OSA and the attenuation of BMD using clinical CT scans. Earlier studies reported considerable variability in BMD between patients^16^, and it is possible that longitudinal assessment of BMD changes could overcome this limitation. Here, we performed a comparison study to explore alterations (in Hounsfield units; HU) in longitudinal abdominal CTs for other indications in OSA patients and no-OSA participants. The study was conducted with enrollees at the Soroka University Medical Center (SMC) from the Clalit Health Services (CHS) group, the largest health maintenance organization in Israel that keeps strict electronic registry of patient records^3,4,18^.

## Results

### Participant characteristics

**Figure 1:**
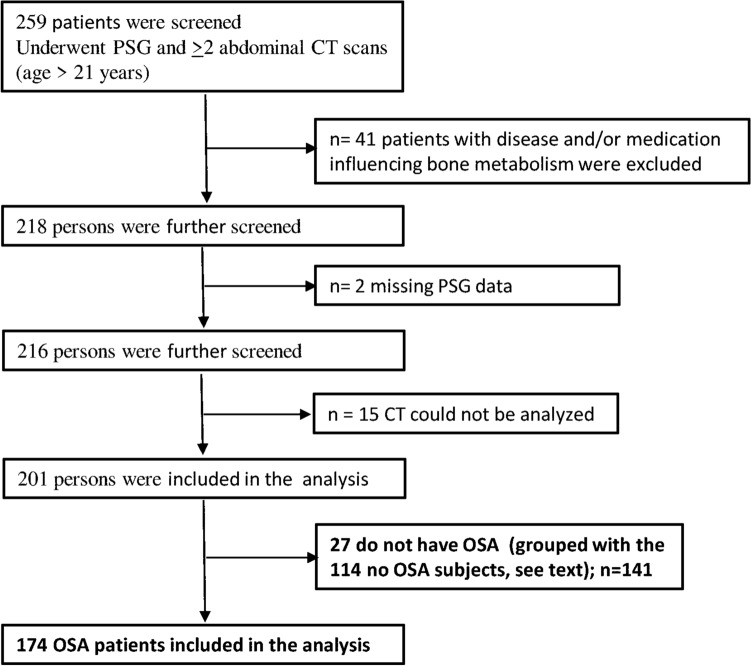
Flow chart of study participants.

174 OSA patients (apnea–hypopnea index, AHI ≥ 5 events/hr) (60.9% men) (Fig. 1) and 141 participants without OSA (79, 56% men) were recruited. No significant differences were found in gender distribution (men/women, 79/62 and 106/68) between the no-OSA and OSA groups, respectively. The prevalence of hypertension and cardiovascular diseases was significantly higher in the OSA group (Table 1, *p* < 0.001). Table 2 summarizes patient characteristics according to OSA severity. Moderate/severe OSA versus mild OSA patients were older (*p* = 0.007), had a higher arousal and awakening index (*p* < 0.05), and a higher ODI (≥ 4%) index (*p* < 0.001). Women with OSA versus no OSA were about 5 years older than women with no OSA (Table 3, *p* < 0.05). Both groups had a similar duration and duration range between their first and second CT scans (Table 3, *p* = 0.903).‏ The 12 thoracic vertebra BMD correlated with the first lumbar vertebra BMD on the first r = 0.863, (*p* < 0.001) and the second CT scan r = 0.868 (*p* < 0.001), respectively (Supplementary Fig. 1).

**Table 1:** Associate morbidity.

	No OSA	Mild OSA	Moderate/severe OSA	p-value
n	141	77	97	
CVD	28%	39%	51.6%	< 0.001
HTN	33.4%	54.6%	54.6%	< 0.001

*CVD* cardiovascular disease, *HTN* hypertension.

p-value was determined by chi-square test.

**Table 2:** Polysomnographic characteristics of obstructive sleep apnea patients.

	Mild OSA	Moderate/severe OSA	p-value
N	77	97	
AHI (events/h)	10.4 ± 2.7	35.7 ± 19.1	0.001
Men	53.3%	67%	0.11
Age (years)	58.9 ± 12.9	63.8 ± 9.9	0.007
BMI (kg/cm^2^)	30.1 ± 6.5	35.6 ± 32.2	0.111
Ar index (events/h)	26.8 ± 27.6	35.0 ± 21.3	0.037
Sleep efficiency (%)	82.8 ± 10.1	81.9 ± 13.8	0.657
Wake O_2_	95.7 ± 2.8	95.7 ± 3.0	0.866
T_90_ (%)	8.8 ± 14.8	15.1 ± 23.1	0.09
DI (events/h)	11.9 ± 5.3	29.4 ± 16.7	0.001
ESS (score)	9.3 ± 6.4	8.8 ± 9.7	0.774

*AHI* apnea–hypopnea index, *Ar index* Arousal and Awakening Index, *BMI* Body Mass Index, *ESS* Epworth Sleepiness Scale, *T*_*90*_ percent sleeping time in which oxygen saturation was below 90%, *DI* desaturation index ≥ 3%. Values are mean ± SD.

*p*-value was determined by an unpaired Student’s t-test.

**Table 3:** Vertebrae bone mineral density.

	All		Women		Men	
	No OSA	OSA	No OSA	OSA	No OSA	OSA
Number of vertebrae	202	238	84	100	118	138
Age (years)	58.8 ± 12.5	61.6 ± 12**	58.3 ± 12.1	63.6 ± 11.2**	59.2 ± 12.7	60.3 ± 12.3
Mean TBS (day)	1309	1298				
TBS (rang)	31–3125	37–3207				
F- BMD (HU)	136.3 ± 3.1	134.8 ± 2.9^+^	138.0 ± 5.5	134.0 ± 4.7^+^	135.0 ± 3.9	135.3 ± 3.7
S- BMD (HU)	129.0 ± 3.1^+^	122.0 ± 2.8^+^	132.4 ± 5.2^+^	120.7 ± 4.6^+^	126.7 ± 3.8^+^	122.9 ± 3.6^+^
BMD DIFF (HU)	− 7.5 ± 1.5	− 12.8 ± 1.2^##^	− 7.8 ± 2.6	− 13.4 ± 1.9	− 7.2 ± 1.7	− 12.4 ± 1.54^#^

Bone mineral density of combined 12 thoracic (T12) and first lumbar (L1) vertebrae; Data include participants who were not administered a contrast agent; *OSA* obstructive sleep apnea (apnea–hypopnea index ≥ 5 events/h), *TBS* time duration between scans, *F* first scan, *S* second scan, *DIFF* difference in HU between second and first CT scans, negative sign indicates a loss in BMD, *HU* Hounsfield unit. Values are mean ± SD for age and SEM for the remaining parameters.

^#^*p* < 0.05, ^##^*p* < 0.01 first scan vs. second scan. Statistical differences were determined by a two-tailed t-test.

^+^*p* < 0.01, no-OSA vs. OSA, statistical differences were determined by a 2-way repeated measurements ANOVA.

### Analysis of BMD including subjects who received a contrast agent

174 OSA patients and 141 participants without OSA were analyzed. Of them n = 55 OSA and n = 40 non-OSA patients, received a contrast agent*.* The BMD of OSA versus no OSA was significantly decreased (F = 5.9, *p* < 0.05), and in both groups, BMD significantly decreased over time (F = 10.67, *p* < 0.001) (Supplementary Table S1). The difference in BMD (i.e., the BMD in the second scan minus the BMD in the first scan) was significantly attenuated by about 6.6 HU in the OSA versus no-OSA group (Supplementary Table S1, *p* < 0.01). Women with OSA compared to those without OSA had a low BMD of about 11–17 HU (F = 6.84, *p* < 0.01), and over time, the BMD was attenuated in both groups (F = 4.23, *p* < 0.05). Over time, the BMD in men was significantly attenuated in both groups (F = 6.49, *p* < 0.05). No significant changes were found in the BMD between groups in men (F = 0.59, *p* = 0.44). A BMD difference was attenuated significantly more in men with OSA versus the no-OSA group by 7 HU (Supplementary Table S1**,** *p*** < **0.01).

### Analysis of BMD excluding subjects who received a contrast agent

**Figure 2:**
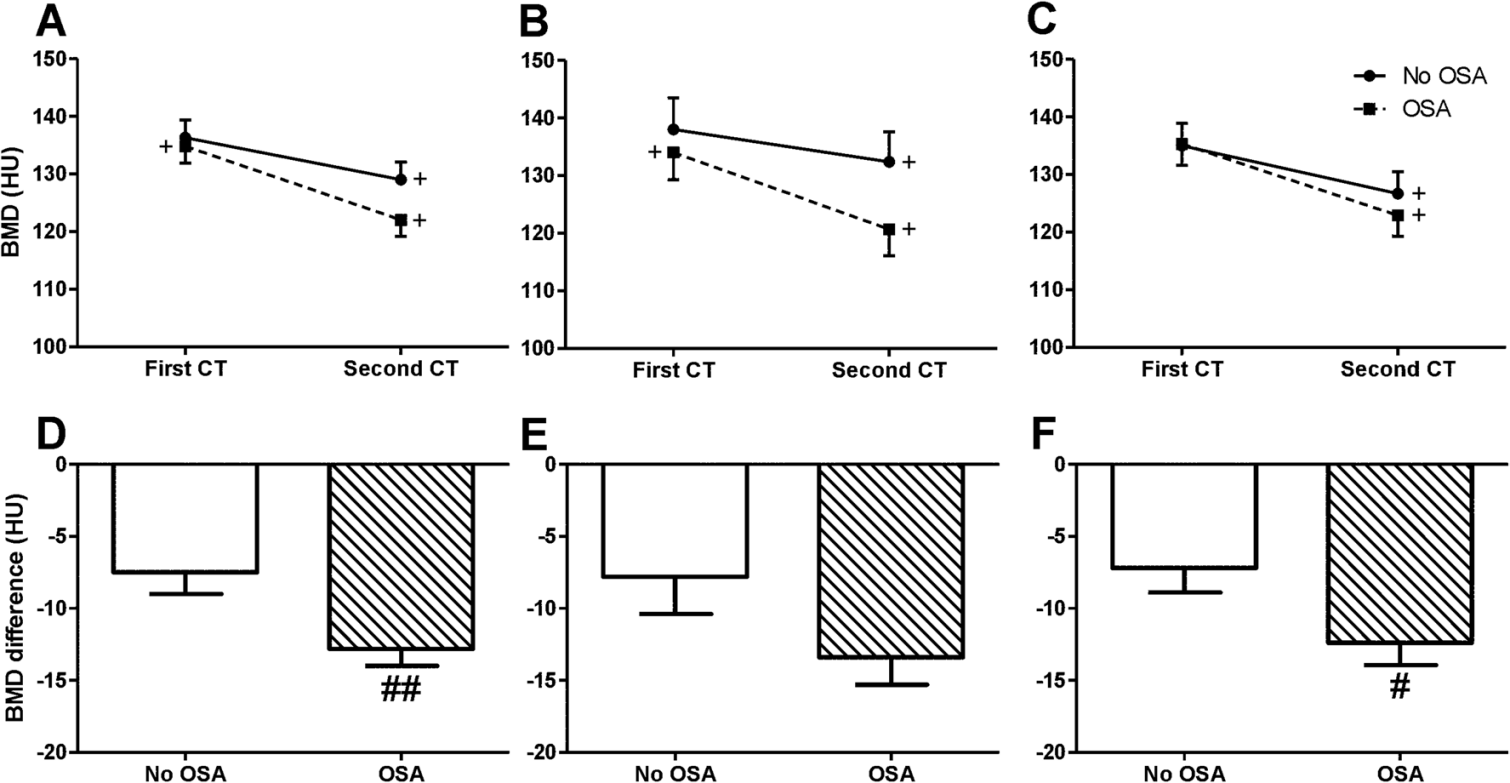
Vertebrae bone mineral density. (**A**) Vertebra BMD for the entire group, (**B**) women’s BMD, (**C**) men’s BMD, (**D**) DIFF for the entire group, (**E**) DIFF for women, (**F**) DIFF for men. Data showing mean BMD of the 12 thoracic vertebrae and first lumbar vertebra; *BMD* bone mineral density, *DIFF* difference in HU between the first and second CT scans. *HU* Hounsfield units, *OSA* obstructive sleep apnea. ^#^*p* < 0.05, ^##^*p* < 0.01 first scan vs. second scan BMD. Statistical differences were determined by a two-tailed t test. ^+^*p* < 0.01, no-OSA vs. OSA, statistical differences were determined by a 2-way repeated measures ANOVA.

In all subjects (n = 119 OSA and n = 101 non-OSA), BMD was significantly attenuated over time in both groups (Table 3, F = 11.03, p < 0.001). BMD was significantly attenuated in the OSA versus no-OSA group (Fig. 2, F = 5.54, *p* < 0.05), and the BMD difference decreased by 5.3 HU more in the OSA group (*p* = 0.01). Decreased BMD was found over time in women from both groups (F = 5.21, *p* < 0.05), and it was attenuated in the OSA versus no-OSA group (F = 7.50, *p* < 0.01). Men with OSA BMD measurements that were similar to the no-OSA group (F = 0.54, *p* = 0.46), and over time, BMD was attenuated in both groups (F = 5.83, *p* < 0.05**)**.

### Factors associated with BMD alterations

BMD was negatively correlated with age r = − 0.49 (*p* < 0.01) and r = − 0.43 (*p* < 0.01) for OSA and no-OSA, respectively. No correlation was found between BMD (or a BMD difference) and polysomnography (PSG) findings such as in the ODI, apnea–hypopnea index, and arousal and awakening indices. The presence of cardiovascular diseases in the OSA versus non-OSA group was associated with lower BMD 127.4 ± 4.7 (HU) versus 139.8 ± 4.2 (HU) (*p* = 0.04) and 125.2 ± 3.9 (HU) versus 142.2 ± 7.9 (HU) (*p* = 0.01), respectively. Hypertension in the OSA versus no-OSA groups was associated with low BMD 126.9 ± 4.3 (HU) versus 142.2 ± 4.6 (HU) (*p* = 0.01) and 129.7 ± 5.4 (HU) versus 144.4 ± 5.3 (HU) (*p* = 0.05), respectively. Neither cardiovascular diseases nor hypertension affected the BMD difference (data not shown).

Supplementary Table S2 shows the multivariate linear regression of determinants of BMD and BMD difference alterations, including subjects who were administered an enhancement agent. Age (β = -1.8 HU, *p* < 0.05) and the presence of OSA (β = − 7.7 HU, *p* < 0.029) were associated with BMD alteration after controlling for the covariates of gender, enhancement agent, and cardiovascular diseases. Age (β = 0.15 HU, *p* < 0.05), OSA (β = − 5.99 HU, *p* < 0.01), and enhancement agent (β = -9.07 HU, *p* < 0.01) were associated with alterations in BMD differences after controlling for the covariates of gender and cardiovascular disease.

Tables 4 exhibits the univariate and multivariate linear regression of determinants of BMD and BMD difference alterations, excluding subjects who received an enhancement agent. Univariate regression indicated that age and cardiovascular diseases (*p* < 0.01) were associated with BMD alterations, and OSA was associated with BMD difference alterations (*p* < 0.01). A multivariate regression indicated that age (*p* < 0.05) and OSA (*p* < 0.01) were associated with alterations in BMD after controlling for gender and cardiovascular diseases. Age (*p* < 0.01), OSA (*p* < 0.05), and cardiovascular diseases (*p* = 0.05) were associated with alterations in BMD differences after controlling for gender.

**Table 4:** Univariate and Multivariate linear regression model on BMD and BMD difference alteration.

	Univariate model		Multivariate model	
	β	95%-CI	β	95%-CI
**Bone mineral density**				
Age (years)	− 1.82	− 2.14 up to − 1.50**	− 1.55	− 1.91 up to − 1.17**
Gender (M/F)	1.95	− 7.20 up to 11.12	8.08	− 0.53 up to 16.7
OSA (yes/no)	− 6.94	− 15.90 up to 2.00	− 12.80	− 21.40 up to − 4.19 **
CVD (yes/no)	− 15.72	− 24.91 up to − 6.52**	4.96	− 4.56 up to 14.49
**Bone mineral density difference**				
Age (years)	0.12	− 0.03 up to 0.29	0.21	0.032 up to 0.38**
Gender (M/F)	0.88	− 3.27 up to 5.04	− 1.59	− 2.54 up to 5.74
OSA (yes/no)	− 5.29	− 9.33 up to − 1.25**	− 4.62	− 8.78 up to − 0.47*
CVD (yes/no)	− 3.80	− 8.03 up to 0.42	− 4.52	− 9.13 up to − 0.01*

*β* unstandardized β, Bone Mineral Density (BMD) of combined 12 thoracic vertebrae and first lumbar vertebra scans. Data include participants who were not administered a contrast agent, *OSA* obstructive sleep apnea diagnosis (apnea–hypopnea index ≥ 5 events/h), *CVD* cardiovascular disease, *F* females, *M* males.

*p = 0.05, **p < 0.01.

## Discussion

A limited number of studies have explored the association between OSA and vertebra BMD using clinical CT scans. To the best of our knowledge, this is the first longitudinal study reporting BMD alterations in OSA using real-life clinical records. OSA is associated with BMD alterations and differences in this alteration independent of age, cardiovascular disease, and/or contrast agent. Due to the retrospective nature of our study, we do not know the causality or mechanism between OSA and BMD. Further exploration is required to explore the mechanism that leads to BMD loss in OSA and its clinical implications, such as vertebra-depressed or femoral neck fractures.

We analyzed BMD following statistical adjustments or exclusion of participants who were administered a contrast agent. BMD decreased more in the OSA group relative to the no-OSA group. Administration of a contrast agent to the same patients in a single session may enhance the trabecular space and affect HU in the range of 11–18; however, considerable variability between patients was observed^19^. Recently, after analysis of a large retrospective cohort of 20,374 CT scans, Jang et al.^16^ concluded that it is debatable whether such differences in HU would be meaningful enough in the setting of optimistic screening.

We found lower BMD in our OSA patients, suggesting a higher risk for osteoporosis. Several clinical studies support our findings that OSA is associated with low bone BMD and fractures^5,6,9^. Interestingly, we found no association between BMD and the oxygen desaturation index (ODI), arousals and the awakenings index, or the apnea–hypopnea index. This finding suggests that classical PSG parameters cannot predict BMD alterations in OSA. Further exploration is required to explore serum markers of bone resorption and sympathetic activity associated with BMD loss in OSA^7,8^. Supporting our findings, in a cross-sectional study of 115 obese men and women with OSA, Mariani et al.^20^ also found a lack of association between the AHI and BMD. Hamada et al.^17^ found that only an alveolar–arterial oxygen pressure difference in OSA was associated with alterations in BMD and that these alterations were not associated with the apnea–hypopnea index, oxygen saturation, or arousals and the awakenings index. Cohort studies found that hypoxia—the hallmark of OSA—was associated with an increased risk for falls, incidents of fractures^21^, lower back pain associated with lumbar spondylosis^22^, and osteoporosis^23^. Sleep fragmentation can affect bone resorption via increased sympathetic tone and/or hormonal factors^7,24–27^. In the current study, cardiovascular diseases and/or hypertension negatively affected BMD and BMD differences in both groups. It is possible that increased age accompanied with cardiovascular diseases, and possibly undiagnosed OSA, could have contributed to BMD in the controls^7,8^. It is also possible that the sympathetic overdrive associated with cardiovascular diseases^28,29^ contributed to MBD loss in both of our study groups, as orexin plays a key role in sleep homeostasis and sympathetic activity, as well as through orexin receptor 1 on bone mass^30^. We recently found that enhanced orexin in chronic upper airway obstruction (in a rat model for OSA) can contribute to inadequate sleep and bone mass loss^32,32^.

Measurement of BMD by dual-energy x-ray absorptiometry revealed an association between OSA and alterations in bone metabolism/architecture^21,33–39^. However, such absorptiometry is limited in measuring alterations in a large body mass index^40–42^. Moreover, confounding information is available regarding the association between OSA and bone health using this type of scanning^20,34–36,38–40^. Because OSA is associated with overweight and obesity^1^, BMD values determined by dual-energy x-ray absorptiometry may be misleading (5–50% in several studies)^41,42^. Thus, further studies are required to reinforce our findings by measuring dual-energy x-ray absorptiometry and bicameral markers at the same time as CT scans are performed.

### Study strengths and limitations

One of the strengths of our study is its “real-life” conditions with no research intervention. We analyzed CT scans for a multitude of conditions and indications not related to OSA of adults referred for OSA diagnosis. All enrollees had free access to medical services^43^. We included enrollees of the largest health maintenance organization in Israel (about 53% of the Israeli population) that keeps strict electronic registry of patient records^3,4,18^. Since physicians are paid per patient by a capitation fee once every three months, they have no economic incentives to prevent or deter patients from medical services^3,4,18,44^. Our study does have some limitations due to its retrospective single-center nature. The non-OSA group could not be considered “normal healthy” since the participants in this group were randomly selected from a large database (of over one million patients during the study period) of ambulatory CT scans in a medical arena. We also were not permitted to contact our participants to obtain additional medical information regarding their BMI, type 2 diabetes, and snoring due to legislation protecting patient confidentiality^43^, limiting the ability to adjust our model to this covariate. According to our medical registry, the no-OSA participants did not have a PSG study history; however, due to poor awareness of sleep-disordered breathing, we cannot rule out the possibility that some may have had unrecognized OSA^45^.

## Conclusions

To the best of our knowledge, this is the first longitudinal study that reports BMD alterations in OSA using real-life clinical records. OSA was associated with alterations in BMD after controlling for age, gender, and cardiovascular diseases. Further studies are needed regarding clinical implications of BMD alterations in OSA, such as vertebra-depressed or femoral neck fractures.

## Methods

### Setting

A retrospective comparison study was conducted at the Sleep–Wake Disorders Center and Imaging Institute at SMC, in which all patients were enrollees of Clalit Health Care Services (CHS). According to the Israeli National Health Insurance Law^43^, all enrollees had free access to medical services, and physicians had no economic incentives to prevent or deter patients from either a PSG study or CT examination^3,4,18^.

This was a retrospective study, and a waiver for informed consent was obtained from the Institutional Review Committee of the SMC. All methods were performed in accordance with Israeli regulations. The Institutional Review Committee of the SMC approved the study protocol (protocol number SOR-20-0250).

### Study protocol

We retrospectively searched the SMC database from June 2010 until September 2020 for all adult patients who underwent an overnight PSG, and performed at least two abdominal CTs for a multitude of conditions and indications not related to OSA. The two CT scans of each participant were done under similar settings. Prior to the PSG, all patients completed a self-administered questionnaire that included sleep habits, clinical history, and the Epworth Sleepiness Scale questionnaire^46^. On the study day, participants were advised to maintain their sleep–wake routine and to avoid consumption of caffeine and soft drinks. The comparison group (no-OSA) were selected randomly from the SMC database that contained over one million abdominal CT scans (during the study period) and were matched by gender with OSA patients.

### Study groups

We identified 259 patients (aged 24 through 85 years) who had undergone an overnight PSG and had at least two ambulatory abdominal CT examinations for conditions not related to OSA. All subjects had “typical” symptoms of OSA and had been referred by an otolaryngology surgeon, pulmonologist, or neurologist. According to our database, none of the no-OSA group had a history of sleep problems and nor had done a PSG study.

Excluded from both groups were patients with any disease that influenced bone metabolism or who were receiving medicine that could influence bone metabolism (n = 41, Fig. 1) such as: chronic obstructive pulmonary disease, genetic disorders, cancer, autoimmune disorders, chronic liver disease, chronic renal insufficiency, musculoskeletal and connective tissue disorders, lumbar surgery, malabsorption disease, fibromatosis, unspecified anticonvulsants, and endocrine disorders. Also, excluded were those undertaking supplemental therapy with calcium or vitamin D or who needed a wheelchair for mobility. We further excluded patients receiving the following medications: oral corticoids, hormone replacement or osteoporosis therapy, proton pump inhibitors, and anticonvulsant and anticoagulant drugs. Of the remaining 280 patients, two subjects had missing PSG data, and in 15 patients, CT data could not be analyzed (the scan was not performed at 120 kV). 201 patients were included in the analysis, and of these, 27 did not have OSA (an AHI < 5 events/hr). Therefore, the OSA group comprised 174 patients.

CT examinations of 124 non-OSA patients were analyzed and, of these, data from 10 CTs could not be analyzed (examination not performed at 120 kV). We grouped 27 PSG patients who tested negative to OSA (AHI < 5 events/h) with the 114 subjects in the comparison group; the no-OSA group included 141 subjects.

Medical diagnoses retrieved from the SMC database are documented only by physicians using the International Classification of Diseases, Ninth Revision (ICD-9) code. This database contains > 99% of all patient diagnoses. We reviewed the following diagnosed cardiovascular diseases [codes 410–414, 426–438, 443] and hypertension [codes 401–405]. A self-administered questionnaire assessed the Epworth Sleepiness Scale, in which a higher score indicates a higher level of sleepiness^46^.

### PSG study

Data were acquired using a commercially available sleep monitoring system (Viasys, SomnoStar Pro; Yorba Linda, CA, USA or SomniPro 19 PSG; Deymed Diagnostic, Hronov, Czech Republic), as previously described by our laboratory. The overnight PSG included recordings of an EEG (C3/A2, C4/A1, and O2/A1, O1/A2, according to the international 10–20 system), EOG, EMG, and ECG electrode abdomen and chest effort belts to measure respiratory activity, and an oxygen saturation sensor (SomniPro 19 PSG, Deymed Diagnostic, Hronov, Czech Republic). Scoring was done by a trained technologist and reviewed by one of the investigators (A.T.). Arousals and awakenings were scored using the American Sleep Disorders Association (ASDA) assessment^47^. The AHI was defined as the sum of all obstructive and mixed apneas, plus hypopneas associated with a ≥ 30% reduction in airflow and either ≥ 4% oxygen desaturation or electroencephalographic arousal, divided by the hours of total sleep time^47^. In addition, the percent of sleeping time in which oxygen saturation was below 90% (T_90_) was calculated. OSA severity was defined as AHI of 1–4.9 events/h, AHI of 5.0–14.9 events/h, or AHI ≥ 15 events/h were considered as no-OSA, mild OSA or moderate/severe OSA, respectively.

### BMD measurement of the vertebral bone

**Figure 3:**
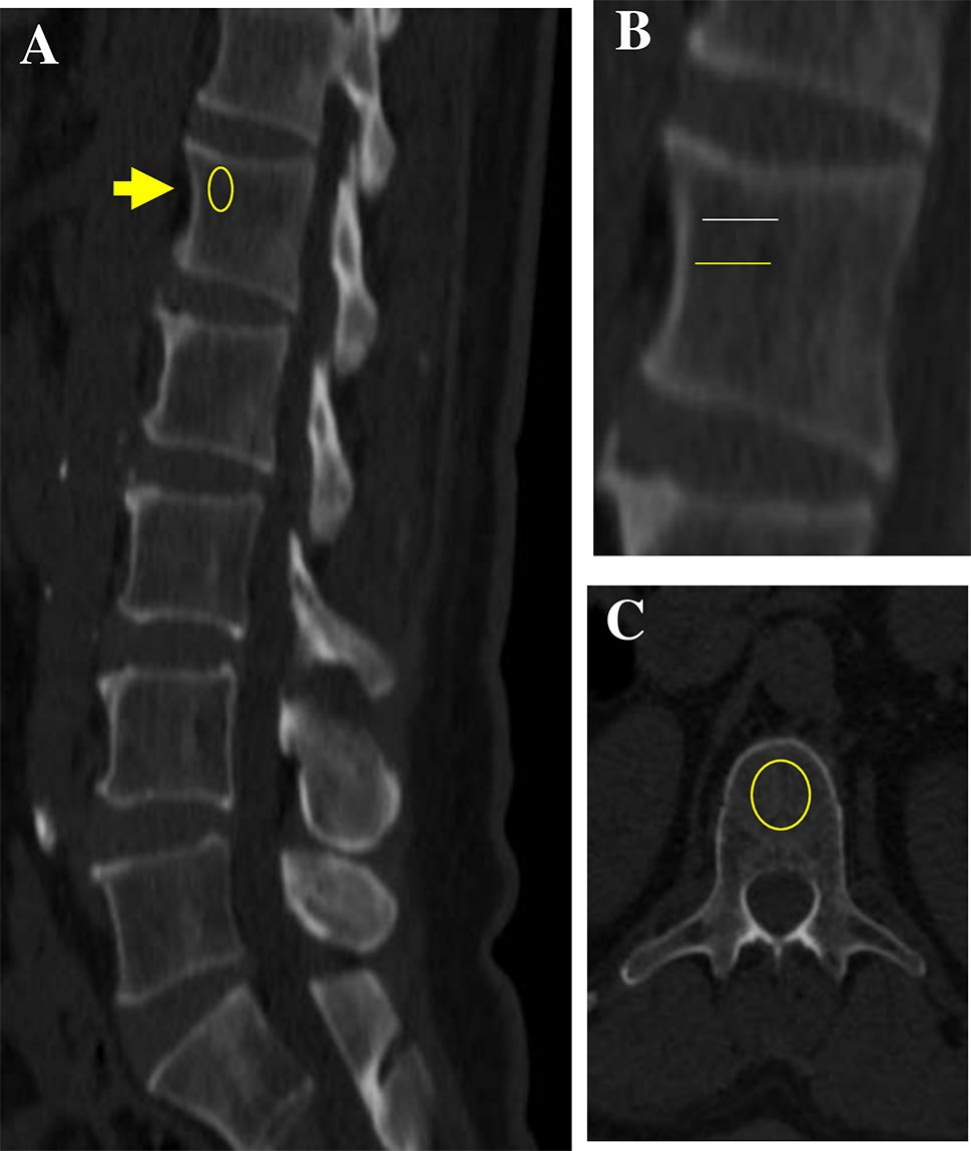
Example of unenhanced computed tomography (CT) images of the spine of a 59-year-old woman. (**A**) Bone window showing the first lumbar vertebra level (arrow) and oval yellow ring shows the ROI, (**B**) Magnified view of the first lumbar vertebra and region of interest. The yellow midline line matches the automated level; the white line reflects the standard manual level. (**C**) First lumbar vertebra axial image; oval yellow ring shows the region of interest. CT examination was performed at a constant peak voltage of 120 kV.

We used 1-mm thick abdominal sections from CT examinations, obtained during ambulatory or emergency room visits, using a Siemens SOMATOM Definition AS + Scanner (Siemens Healthcare GmbH, Erlangen Germany) or a Philips Brilliance ICT scanner (Haifa, Israel). We retrospectively accessed the CT examinations at a constant peak voltage of 120 kV with a variable mAs tube. We included CT scans with or without a contrast agent (n = 76 no-OSA and n = 90 OSA), and evaluated vertebral BMD on a standard radiology picture archiving and communication (PACS) system workstation, with images viewed in bone windows, i.e., gray-scale assignment of the image display, to emphasize bone without the influence of alteration/BMD values (Fig. 3)^16,48^. We included examinations either with or without an intravenous enhancement agent. We assessed vertebral BMD by placing a single oval click-and-drag region of interest (ROI) in an axial and sagittal slice over an area of vertebral body trabecular bone and then measured CT alteration in HU, with lower HU (lower alteration) representing less-dense bone, at each of the T12 and L1 levels. On the axial images of the selected slice—the superior part of the vertebra—the elliptical ROI was encompassed manually as the largest possible area at the anterior portion of each vertebral body, and in the sagittal plane, we focused on the upper anterior part of the vertebra in order to avoid the Dense Tracecular zone. The mean CT scan density of the ROI was measured. We avoided placing the ROI near areas that would distort the BMD measurement (focal heterogeneity or lesion, posterior venous plexus, compression fracture, and artifacts). The two CT scans of each participant were performed under similar settings, and a CHS engineer from the Biomedical Engineering Department calibrated the CT scanners routinely according to the manufacturer’s instructions using an American College of Radiology-accredited phantom. BMD was expressed in milligrams per milliliter of hydroxyapatite^16^.

### Statistical analysis

Statistical analyses were performed using R Statistical Software, version 3.5.2 (Foundation for Statistical Computing, Vienna, Austria). We compared the proportion of cardiovascular diseases and hypertension between patients with and without OSA using a Pearson chi-square test. Age, body mass index, the Epworth Sleepiness Scale, the arousal and awakening index, sleep efficiency, oxygen desaturation index (> 4%), and the percent of sleeping time in which oxygen saturation was below 90% were compared between patients with mild and moderate/severe OSA using a Student's t-test. The first and second BMD measurements and the difference between the two BMD (i.e., BMD on the second scan minus BMD on the first scan) measurements were compared between the OSA and no-OSA groups using a Student's t-test. We further evaluated the difference within the two measurements between the OSA and no-OSA groups using a two-way repeated measurements ANOVA. We calculated the study power for two different sample sizes: the first- all patients in the study, including patients who received contrast agents on imaging (OSA patients- 174, non-OSA patients-141; OSA vertebrae- 348, non-OSA vertebrae- 282). The second—patients who did not receive contrast agents on imaging only (OSA patients- 119, non-OSA patients -101, OSA vertebrae- 238, non-OSA vertebrae- 202). The calculated power to detect a difference of 1 HU in BMD between vertebrae of all patients with and without OSA with a basic mean BMD of 131 HU, a standard deviation of 2.6 HU and α = 0.05 was 99.9%. Furthermore, the calculated power to detect a difference of 1 HU in BMD between vertebrae of patients with and without OSA for which contrast enhancement was not used with a basic mean BMD of 129 HU, a standard deviation of 3.1 HU and α = 0.05 was also 99.9%. To assess the independent association between OSA and the first measurement BMD and the BMD difference between the two measurements, we used a multivariate linear regression adjusted for patient age, gender, and diagnosis of cardiovascular diseases. The beta values, including the 95% Confidence Interval (CI) and p values for each variable, were calculated. Null hypotheses were rejected at the 5% level.

## Supplementary Information

Supplementary Information.
