# Supplementary material for: Bone mineral density alteration in obstructive sleep apnea by derived computed tomography screening
Source: Sci Rep. 2022 Apr 19;12:6462. doi: 10.1038/s41598-022-10313-w (PMC9018731; doi:10.1038/s41598-022-10313-w)
Supplement: Supplementary file 1 — Supplementary Information. [file 41598_2022_10313_MOESM1_ESM.pdf]

Supplementary Information

Bone Mineral Density Alteration in Obstructive Sleep Apnea by Derived Computed Tomography Screening

Sharon Daniel, Yafit Cohen-Freud, Ilan Shelef, Ariel Tarasiuk

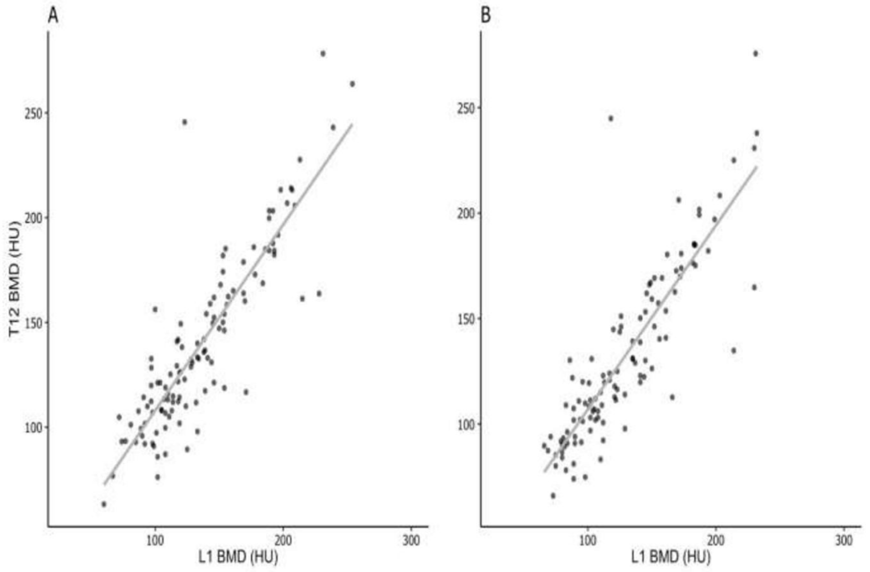

<u>Supplementary Figure 1</u>: Correlation of bone mineral density T12 and L1 vertebrae.

A) First CT examination (r = 0.863, $p < 0.001$); B) Second CT examination scan (r = 0.868, $p < 0.001$); T12 – twelve thoracic vertebrae; L1 – first lumbar vertebrae.

Supplementary Table S1: Vertebrae bone mineral density

	All		Women		Men	
	**No OSA**	**OSA**	**No OSA**	OSA	**No OSA**	**OSA**
Number of vertebrae	282	348	124	136	158	212
Age (years)	59.5 ± 13	61.6 ± 11.5	58.6 ± 12.7	62.8 ±11.2*	60.1± 13.3	60.9 ±11.7
F- BMD (HU)	139.5 ± 2.7	134.9 ± 2.4^{+}	145.1± 4.5	134.4 ± 3.8^{+}	135.3 ± 3.45	135.2 ± 2.9
S- BMD (HU)	131.4 ± 2.6$^{\#}$	121.2 ± 2.8^{+}	137.0 ± 4.3^{+}	120.3 ± 3.6^{+}	127.3 ± 3.3^{+}	121.7± 2.8^{+}
BMD DIFF (HU)	-8.3 ±1.2	-14.8 ±1.1#	-10.0 ± 2.0	-14.2 ± 1.6	-7.0 ±1.5	-15.2 ± 1.4#

BMD – Bone mineral density of combined twelve thoracic (T12) and first lumbar (L1) vertebrae. Data include participants that were administered /or were not administered a contrast agent; OSA – obstructive sleep apnea (apnea–hypopnea index ≥ 5 events/hr); F – first computed tomography scan; S – second computed tomography scan; DIFF – difference in HU (Hounsfield units) between the second and first CT scans; The negative sign indicates a loss of BMD.

Values are mean ± SD for age and SEM for the remaining parameters.

# $p < 0.01$ first scan vs. second scan; differences were determined by a two-tailed t-test.

+ $p < 0.01$, no OSA vs. OSA; statistical differences were determined by a 2-way repeated measurements ANOVA.

Supplementary Table S2: Multivariate linear regression model on BMD and BMD difference attenuation.

	Bone Mineral Density		Bone Mineral Density Difference	
	β	95%-CI	β	95%-CI
Age (years)	-1.80	-2.11 up to –1.49**	0.15	-0.09 up to 0.3*
Gender (F/M)	-6.08	-13.53 up to 1.36	-1.2	-2.60 up to 5.07
OSA (yes/no)	-7.71	-14.99 up to -0.42*	-5.99	-9.83 up to -2.17**
Enhancement agent (yes/no)	7.97	-1.8 up to 17.8	-9.07	-14.23 up to -3.92 **
CVD (yes/no)	1.83	-6.18 up to 9.84	-3.79	-8.02 up to 0.43

β – Unstandardized β; Bone mineral density of combined twelve thoracic vertebra and first lumbar vertebra scans. Data include participants that were administered or not administered with a contrast agent; OSA – obstructive sleep apnea diagnosis (apnea–hypopnea index ≥ 5 events/hr); CVD – cardiovascular disease; F– females; M – males.

* $p = 0.05$, ** $p < 0.01$.
